# Association between Circular RNA CDR1as and Post-Infarction Cardiac Function in Pig Ischemic Heart Failure: Influence of the Anti-Fibrotic Natural Compounds Bufalin and Lycorine

**DOI:** 10.3390/biom10081180

**Published:** 2020-08-14

**Authors:** Julia Mester-Tonczar, Johannes Winkler, Patrick Einzinger, Ena Hasimbegovic, Nina Kastner, Dominika Lukovic, Katrin Zlabinger, Andreas Spannbauer, Denise Traxler, Sandor Batkai, Thomas Thum, Mariann Gyöngyösi

**Affiliations:** 1 Department of Internal Medicine II, Division of Cardiology, Medical University of Vienna, 1090 Vienna, Austria; julia.mester-tonczar@meduniwien.ac.at (J.M.-T.); johannes.winkler@univie.ac.at (J.W.); n1542442@students.meduniwien.ac.at (E.H.); nina.kastner@meduniwien.ac.at (N.K.); dominika.lukovic@meduniwien.ac.at (D.L.); katrin.zlabinger@meduniwien.ac.at (K.Z.); andreas.spannbauer@meduniwien.ac.at (A.S.); denise.traxler-weidenauer@meduniwien.ac.at (D.T.); 2 Institute of Information Systems Engineering, Research Unit of Information and Software Engineering, Vienna University of Technology, 1040 Vienna, Austria; patrick.einzinger@tuwien.ac.at; 3 Institute of Molecular and Translational Therapeutic Strategies (IMTTS), Hannover Medical School, 30625 Hannover, Germany; batkai.sandor@mh-hannover.de (S.B.); thum.thomas@mh-hannover.de (T.T.); 4 REBIRTH Center of Translational Regenerative Medicine, Hannover Medical School, 30625 Hannover, Germany

**Keywords:** cardiac fibrosis, porcine model, cardiovascular disease, bufalin, lycorine, circular RNA, CDR1as

## Abstract

Anti-fibrotic therapies are of increasing interest to combat cardiac remodeling and heart failure progression. Recently, anti-fibrotic circular RNAs (circRNAs) have been identified in human and rodent cardiac tissue. In vivo (rodent) experiments proved cardiac anti-fibrotic effects of the natural compounds bufalin and lycorine by downregulating miRNA-671-5p, associated with a theoretic increase in the tissue level of circRNA CDR1as. Accordingly, we hypothesized that both anti-fibrotic drugs may inhibit focal myocardial fibrosis of the remodeled left ventricle (LV) also in a translational large animal model of heart failure (HF). Domestic pigs were repeatedly treated with subcutaneous injections of either bufalin, lycorine, or saline, (n = 5/group) between days 7–21 post acute myocardial infarction (AMI). At the 2-month follow-up, both bufalin and lycorine led to significantly reduced cardiac fibrosis. Bufalin treatment additionally led to smaller end-diastolic volumes, higher LV ejection fraction (EF), and increased expression of CDR1as of the AMI region. Elevated tissue levels of the circRNA CDR1as in the AMI region of the pig heart correlated significantly with LV and right ventricular EF, LV stroke volume, and negatively with infarct size. In conclusion, we successfully identified the circRNA CDR1as in pig hearts and show a significant association with improved LV and RV function by anti-fibrotic therapies in a translational animal model of HF.

## 1. Introduction

A myocardial infarction inevitably results in a considerable tissue loss. The consequence is an immediate increase in the contractile workload of the remaining ischemia-untouched myocardium. To maintain the left ventricular (LV) function and systemic circulation, series of initially adaptive and subsequently maladaptive changes occur in the chronic phase of ischemic heart disease [1,2,3]. The uneven pressure and volume overload of the ischemia-unaffected opposite LV wall provoke a number of macroscopic and molecular alterations [2,4,5] leading to LV adverse remodeling. Remodeling not only affects the ischemic tissue but also alters gene expression and pathway network changes in non-ischemic regions of the myocardium [5]. While a sterile inflammation of the ischemic tissue may have favorable effect during the early stages of infarction, persistent inflammation contributes to the production of chemokines, profibrotic cytokines [6], and cardiac fibroblast proliferation [7], leading to the formation of a fibrotic scar. However, the fibrosis is not limited to the infarcted area, but it is also present in remote areas as a response to increased wall stress [1]. Key proteins are matrix metalloproteinases (MMPs), capable of degrading the extracellular matrix (ECM) [4]. In relation to fibrosis, the impairment of the collagen type ratios and an increased overall collagen content are particularly relevant [2]. Fibrillar type I and III collagens accumulate in the infarcted heart, triggering myocardial stiffness [8] leading to focal fibrosis during the adverse remodeling process. Previously, an unbiased large natural compound library screen identified cardioprotective and anti-fibrotic substances, such as bufalin and lycorine that were successfully tested in vitro and subsequently in vivo in hypertension-related myocardial hypertrophy and additional fibrosis models in rodents [9].

### 1.1. Natural Compounds Bufalin and Lycorine Possess Anti-Fibrotic Effects

**Figure 1:**
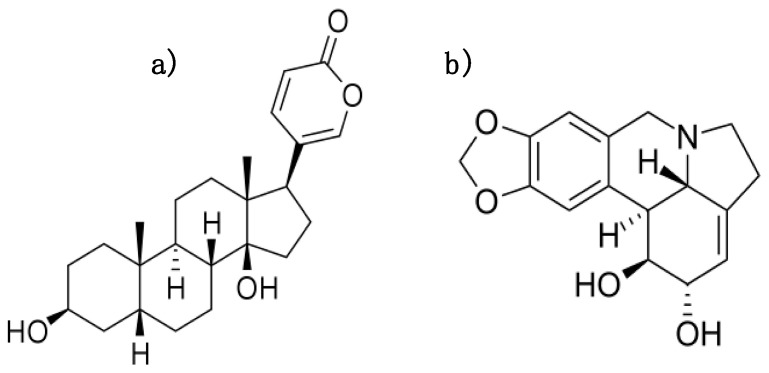
Chemical structure of (**a**) Bufalin and (**b**) Lycorine.

Bufalin (BUF) (Figure 1a) was originally isolated from toad venom. The molecule is a cardiotonic steroid that exhibits a positive inotropic effect on cardiomyocytes [10]. BUF was also studied in vitro and in vivo for different cancer types including hepatocellular carcinoma [11], breast [12], and pancreatic cancer [13].

Lycorine (LYC) (Figure 1b) is a plant alkaloid, found in several species of the *Amaryllidaceae* family. It was previously shown to possess anti-inflammatory, anti-tumor [14], and anti-viral effects [15].

Both BUF and LYC downregulate miRNA-671-5p, a key player in the fibrotic response of murine fibroblasts and human cardiomyocytes [9]. miRNA-671-5p cleaves [16] and represses the anti-fibrotic circular RNA CDR1as, leading to an increase in the mass of the fibrotic tissue [17]. Because of the above-mentioned effects of bufalin and lycorine on miR-671-5p, we examined the expression of circular RNA (CDR1as) and its target miR-671-5p. CDR1as has been studied in a variety of diseases including cancer [18,19], neurological conditions [20], and cardiovascular disease [21]. Due to the non-existent data on the expression of CDR1as in pig hearts [22], we aimed to demonstrate the presence in pig hearts and its effect on post-ischemic cardiac remodeling for the first time.

### 1.2. Aim of the Study

Thus, our study aimed to examine the effect of BUF and LYC on LV adverse remodeling and fibrosis in relation with the deregulation of anti-fibrotic circular RNA CDR1as in a translational model of ischemic heart failure in a pig heart.

We induced closed-chest reperfused acute myocardial infarction in domestic pigs, mimicking human primary percutaneous coronary intervention in ST-elevation myocardial infarction, and administered repeated subcutaneous injections of LYC and BUF or saline solution (SAL), and investigated (1) the LV and right ventricular (RV) function and remodeling parameter and infarct size, (2) fibrosis of the remodeled ventricular wall, and (3) the expression of miR-671-5p, miR-7 and its target CDR1as. Since non-coding RNAs, such as miR-29a, exert an inhibitory effect on excessive fibrotic and collagen gene expression in cardiac fibroblasts [23], we also investigated (4) the expression levels of crucial components of the ECM, including collagen type I, alpha 1 chain (COL1A1), collagen type III, alpha 1 chain (COL3A1), and matrix metallopeptidase 9 (MMP-9) (5), expression of miR-29a, and the fibrotic genes of COL1A1 and COL3A1.

## 2. Materials and Methods

**Figure 2:**
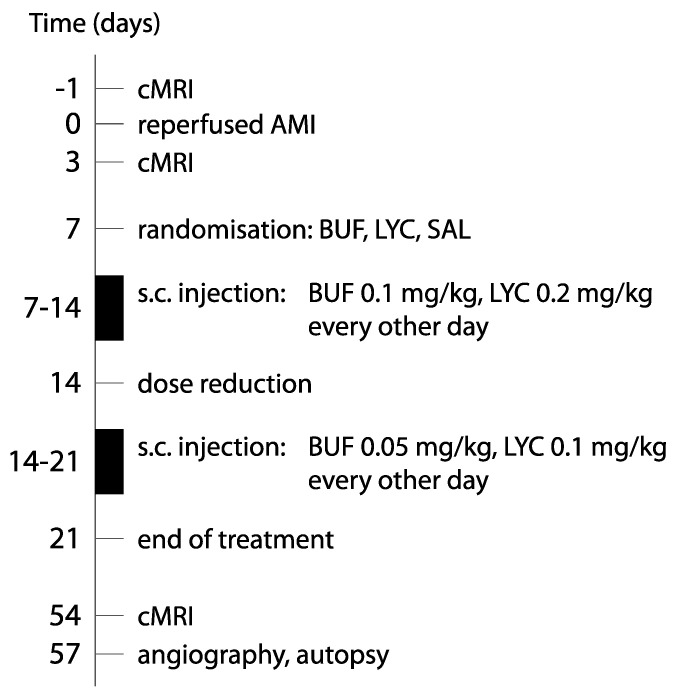
Study design. cMRI = cardiac magnetic resonance; AMI = acute myocardial infarction; BUF = bufalin, LYC = lycorine; SAL = saline; s.c. = subcutaneous injection.

All animal experiments were carried out in accordance with the “Guide for the Care and Use of Laboratory Animals” [24] and were approved by the ethical review committee of the University of Kaposvar (SOI/31/1474-10/2014/KA-1617). Figure 2 shows the study design of the animal experiments.

### 2.1. In Vivo Study

#### 2.1.1. AMI Intervention

After overnight fasting, the animals were sedated with 12 mg/kg ketamine hydrochloride, 1 mg/kg xylazine and 0.04 mg/kg atropine, administered by an intramuscular injection. After surgical preparation of the right arteria femoralis, a 6F introducer was placed, and the animals received 200 IU/kg unfractionated heparin intraarterial. Coronary angiography was performed by using 6F guiding catheter. A balloon catheter (diameter: 2.75 mm, length: 15 mm; Maverick XL Monorail Balloon Catheter, Boston Scientific, Boston, MA, USA) was inserted into the left anterior descending coronary artery (LAD), after the origin of the second diagonal branch. Reperfused acute myocardial infarction was induced by balloon occlusion of the mid LAD for 90 min, with a balloon inflation pressure of 5 atm, followed by the deflation of the balloon, inducing reperfusion. Control angiography confirmed the patency of the LAD. After hemodynamic stabilization, the balloon, guiding wire, catheter, and the introducer were removed, and the artery was sutured. The skin wound was closed in 3 layers, and the animals were allowed to recover.

#### 2.1.2. Follow-Up Investigations and Medical Treatment of AMI

One day before AMI, the animals received a loading dose of clopidogrel (300 mg) and 500 mg aspirin per os. During the 2 months follow-up, a daily dose of 75 mg clopidogrel and 100 mg aspirin was administered per os. To quantify the left ventricular function (LVF) after reperfused AMI, and to measure the size of infarction and microvascular obstruction, cMRI was performed at day 3 post-AMI and at the final 57 days follow-up. The animals were then euthanized with 10 mL intravenous saturated KCl (10%) under general anesthesia.

#### 2.1.3. Treatment Groups

Since we intended to inhibit fibrogensis and adverse remodeling, we started the treatments with BUF and LYC seven days after AMI, the animals being in the downstream phase of the main inflammatory processes. The animals were then randomized into three groups: BUF (*n* = 5, 0.02 mg/kg), LYC (*n* = 5, 0.2 mg/kg), or saline (SAL, *n* = 5, 0.2 mL). The randomization was done according to the computed randomization generator. Treatments were administered every other day for the following 21st days by subcutaneous injection. One animal showed a local inflammatory reaction on the site of bufalin injection; therefore, we reduced the doses to 0.01 mg/kg BUF and 0.1 mg/kg LYC 14 days after AMI. To reduce the psychical stress of the animals due to the repeated injections, we terminated the treatment at day 21 post AMI.

#### 2.1.4. Blood Sampling

Blood samples were collected before induction of AMI, after reperfusion, 3, 14, and 57 days post AMI for the measurements of cardiac biomarkers troponin I (TnI) (baseline, post-reperfusion and at 3-day), NT-proBNP (baseline, at 3-days and 2-month), and liver enzymes (baseline, at 14-days and 2-month) (alanine-aminotransferase, ALT, aspartate-aminotransferase, AST, and alkaline phosphatase, ALP). The porcine normal range for serum Troponin I levels is 0–40 pg/mL, NT-proBNP is 0–180 pg/mL, and 5–40 mU/mL for ALT, 10–45 mU/mL for AST, and 0–380 mU/mL for ALP. The porcine TnI (Abcam, Cambridge, GB), NT-proBNP (Cloud Clone Corp., Katy, TX, USA), ALT (Sigma Aldrich, St. Louis, MO, USA), AST (Sigma Aldrich), and ALP (Abcam) were assesses by commercially available ELISA.

#### 2.1.5. Collection of Tissue Samples and Staining with Haematoxylin–Eosin and Picrosirius for Myocardial Fibrosis

Tissue samples of approximately 150–250 mg were collected from the infarcted and remote (lateral wall) regions of the pig hearts following the autopsy and stored in 5% formaldehyde and RNA Later (Thermo Fisher Scientific, Waltham, MA, USA). For histological proof of LV remodeling, we examined cardiac fibrosis and compensatory cardiomyocyte hypertrophy as evidenced by the increase in cardiomyocyte diameter of the remote lateral wall. The fibrosis was quantified as the percentage fibrotic area in a standard Picrosirius Red stain. The cell diameter was calculated using a standard Haematoxylin–Eosin stain (Supplementary Methods).

#### 2.1.6. Cardiac Magnetic Resonance Imaging with Late Enhancement (cMRI+LE)

cMRI+LE was performed at day 3 and 2-month after infarction under general anesthesia. The acquisition method and methods for calculation of the left and right ventricular parameter (end-diastolic and end-systolic volumes (EDV and ESV), stroke volume (SV), ejection fraction (EF), area at risk, infarct size, and microvascular obstruction) were published elsewhere [25].

#### 2.1.7. RNA Isolation from Tissue, cDNA Synthesis for mRNA and miRNA and Real-Time qPCR mRNA and miRNA

These methods are described in the Supplementary Materials. The primers are listed in Supplementary Table S1.

### 2.2. CDR1as Study in Pig Hearts

To our knowledge, no publication has described a successful detection of CDR1as in the porcine heart. The putative sequence was derived from the NCBI database, by comparing the CDR1as sequence of humans and mice with the potential sequence of CDR1as in pigs. We then designed divergent primers to ensure amplification of the backsplice junction, which can only be found in circular transcripts. Sanger sequencing was performed by splitting the putative CDR1as in smaller parts. In the end, the sequencing results were put together to obtain the full sequence of porcine CDR1as.

#### Sanger Sequencing of CDR1as

To determine if the previously amplified qPCR product possesses a backsplice junction and to check the sequence of porcine CDR1as Sanger sequencing was performed. Therefore, the presumed sequence of CDR1as was split in several parts and qPCR product, and divergent primers were sent to Sanger sequencing (Microsynth). In the end, the sequencing results were assembled to obtain the entire porcine CDR1as sequence.

### 2.3. Statistics

Since no previous data regarding bufalin and lycorine treatment in porcine ischemic heart failure existed, no sample size calculation was done. Statistical analyses were performed using R (version 3.6.2). Data are presented as mean ± standard deviation. Two-way mixed ANOVA was conducted to assess the effect of treatment (between-group independent variable) on transcript expression in two heart regions (repeated-measures independent variable) and the effect of treatment on toxicology marker expression at two time points (repeated-measures independent variable in this case). Tukey’s method detected outliers for several combinations of treatment groups, heart regions, and outcome. Moreover, the normal distribution assumption, which was tested with the Shapiro–Wilks test and is needed for a classical ANOVA, was significantly violated in these groups. Therefore, a robust version on the 20% trimmed means was used, as implemented in the bwtrim function of the R package WRS2 (version 1.0.0) by Wilcox [26]. A *p*-value < 0.05 was considered significant. One-way ANOVA on trimmed means was used for post-hoc analysis (R functions t1way and lincon). Spearman’s rank correlation coefficient was used as measure of association between expression levels of different transcripts. Here, we used robust methods instead of the classic ANOVA because of the existence of (often quite severe) single outliers in many groups. While there is no general optimal solution, in our case, the common recommendation of a 20% trimmed mean seems sensible, because in several groups there is exactly one severe outlier. Graphs were made using R studio (ggplot2) or Rhinoceros 3D and the Grasshopper plug-in (Robert McNeel & Associates, Seattle, DC, USA), respectively. All analyses were performed blinded to the treatment groups, including also cMRI.

## 3. Results

### 3.1. Clinical Observations

The animals tolerated the treatment well. Toxicology investigations including the liver enzymes ALT, AST, and ALP activity showed no signs of liver damage (Supplementary Figure S1). With the exception of isolated ventricular extrasystoles during the coronary occlusion and reperfusion, there was no procedural or post-procedural complication.

### 3.2. Cardiac Biomarkers in the Three Groups

There was no difference between the groups regarding baseline or follow-up TnI or NT-proBNP (Supplementary Figure S2). The changes between baseline and post-AMI and 3d follow-up for TnI and between baseline and 3 days and 2-month follow-up for NT-proBNP did not differ statistically, with a small visual trend towards a larger increase in TnI post-AMI and 3-day FUP in the saline group (Supplementary Table S2).

### 3.3. Analysis of Left and Right Ventricular Function and Infarct Size

**Figure 3:**
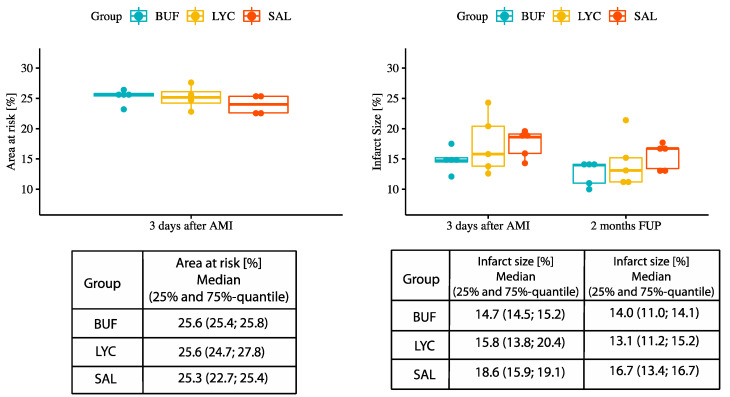
Area at risk (3 days after AMI) and infarct size (3 days and 2 months after AMI) in the BUF, LYC, and SAL-treated groups. Median, 25%-quantile, and 75%-quantile.

Three-day cMRI+LE showed similar area at risk and a trend towards smaller infarct size in BUF group in comparison with the SAL group at 3 days and at 2 month follow-up (*p* = 0.125) (Figure 3).

There was no difference in microvascular obstruction (Supplementary Figure S3) or LV end-diastolic and end-systolic or stroke volumes between the treatment groups.

**Figure 4:**
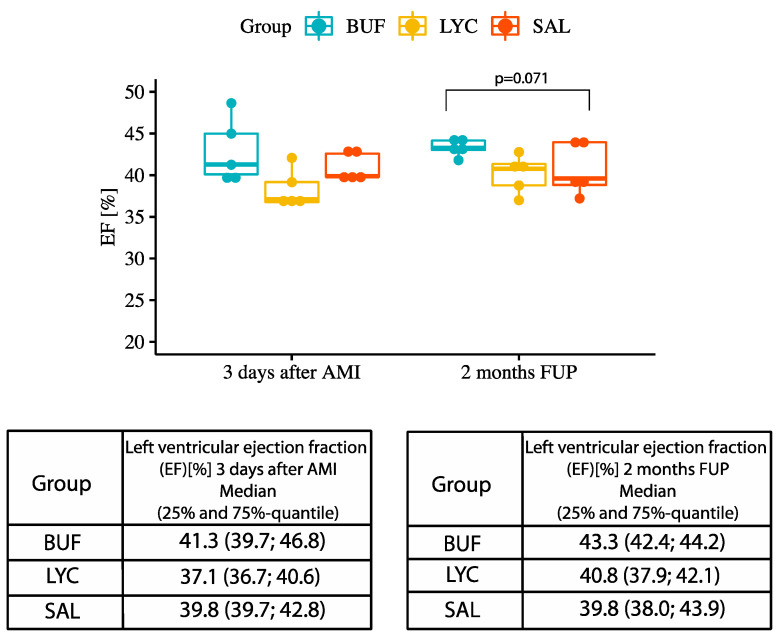
Ejection fraction (EF) 3 days and 2 months after AMI in the treatment groups.

A trend towards higher LV EF was found in the BUF group at 2 months after AMI, as compared with the slight decrease in EF in SAL group (Figure 4). There was no statistically significant difference between the groups regarding 3d cardiac MRI results.

Two-month cMRI-LE revealed no difference between the groups regarding RV EDV, ESV, and SV, but a trend towards better RV EF was found in the bufalin group (Supplementary Figure S4). Changes in LV EF and RV EF of individual animals between 3 days post AMI and at the 2 months FUP are presented in Supplementary Figure S5.

### 3.4. Histological Analysis of the Myocardium

**Figure 5:**
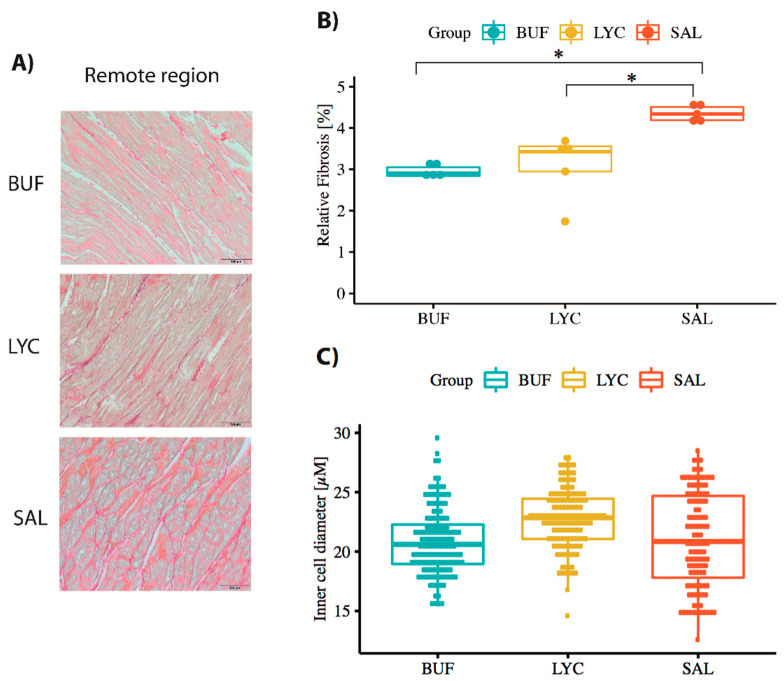
Cardiac fibrosis in the remote region using Picrosirius red stain (PSR) and cardiomyocyte cell diameter calculation. (**A**) Representative histological picture of remote myocardial area of all groups, staining with picrosirius for fibrosis. (**B**) Quantitative analysis of fibrosis (* indicate *p* < 0.05 between BUF/LYC vs. SAL groups). (**C**) Myocyte diameter of the remote myocardial area.

Picrosirius red staining (PSR) showed significantly less fibrosis in both bufalin and lycorine groups as compared with the SAL group in the remote myocardial area (Figure 5A,B). There was no difference between the groups regarding the cardiomyocyte diameter, where 130 cells/group were measured for their diameter (Figure 5C).

### 3.5. RNA Analyses

Real-time qPCR was performed to determine the relative gene expression of COL1A1, COL3A1, MMP-9, and miR-29a in the infarcted region and remote region of the MI and between the treatment groups.

**Figure 6:**
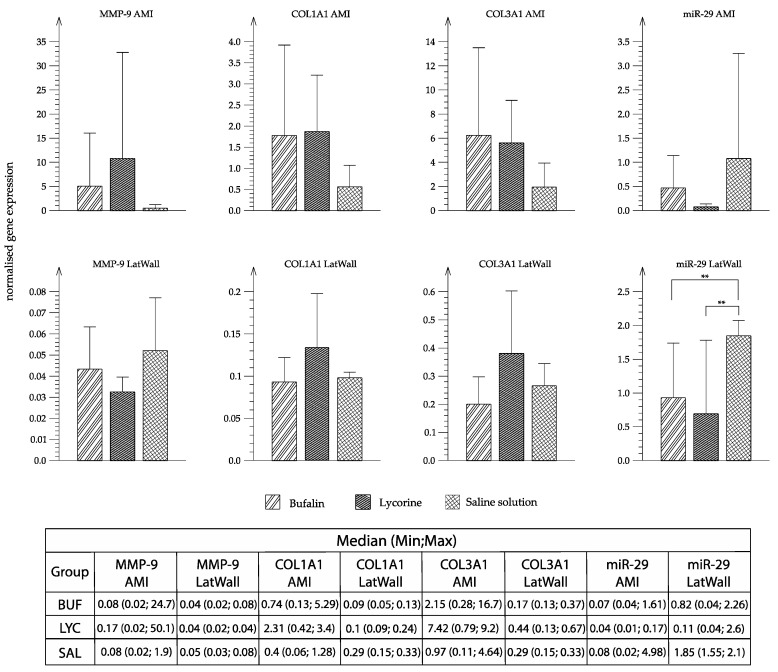
Gene expression of MMP-9, COL1A1, COL3A1, and miR-29. Evaluation of normalized gene expression of MMP-9, COL1A1, COL3A1, and miR-29 in the infarct region (AMI) and lateral wall using qPCR. RNA was isolated using column-based chloroform extraction and reverse transcribed into cDNA. The geometric mean of ACTB and GAPDH was used as reference. For miR-29, let-7a served as reference. Vertical bars show the average of each group, error bars represent standard deviation. ** indicate *p* < 0.01. Each group: *n* = 5, the table shows median (min,max) of MMP-9, COL1A1, COL3A1, and miR-29 of each treatment group.

Except for miR-29, no significant differences between treatment groups were detected by two-way mixed ANOVA. Figure 6 shows a trend to up-regulation of MMP-9 and COL1A1 and COL3A1 in the AMI region and also in the remote lateral wall of the LV both in the BUF and LYC groups compared with placebo. Interestingly, miR-29 was downregulated in the lateral wall in both BUF and LYC groups.

### 3.6. Detection of CDR1as in Pig Hearts and Its Role in Fibrosis

**Figure 7:**
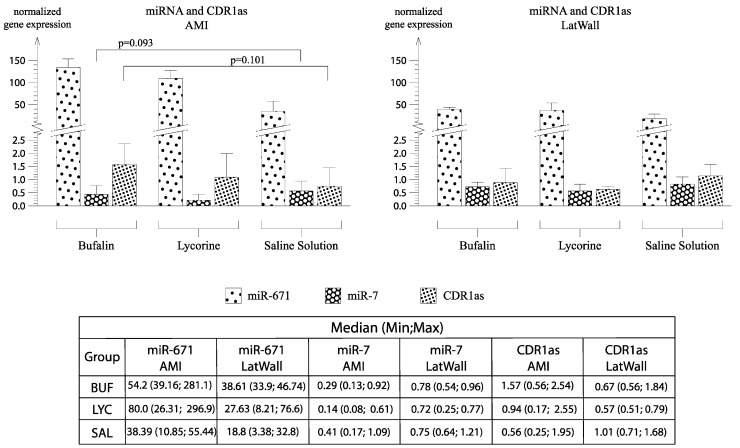
Normalized gene expression of miR-671, miR-7, and CDR1as in the treatment groups in the infarct and remote region of porcine hearts. The table shows median (min;max) of miR-671, miR-7, CDR1as of each treatment group.

A previous study showed that bufalin and lycorine repress miR-671, which is crucial in the fibrotic response of murine fibroblasts and cardiac tissue of aortic stenosis patients [9]. This miRNA was shown to be a potential biomarker in heart failure patients compared to healthy controls [27]. miR-671 is regulated by the circular RNA CDR1as [28]. CDR1as is a circular RNA known for its binding capacity to miR-7 [29]. Interestingly, CDR1as was shown to act as a miR-7 sponge in myocardial cells in mice [21]. Using RNA-Sequencing, a previous study failed to detect CDR1as in pigs [22]. Here, we wanted to see if CDR1as is present in the infarcted pig hearts and how its interaction is with its targets miR-671 and miR-7. Figure 7 shows normalized gene expression of miR-671, miR-7, and CDR1as. Two-way mixed ANOVA showed no significant effect of the treatment groups but with a trend towards higher expression of CDR1as and downregulation of miR-7 in the infarcted area. Additionally, there was a significant difference of miR-7 expression between AMI region and lateral region, if the groups were pooled (F(1, 5.927) = 13.269, *p* = 0.011).

**Figure 8:**
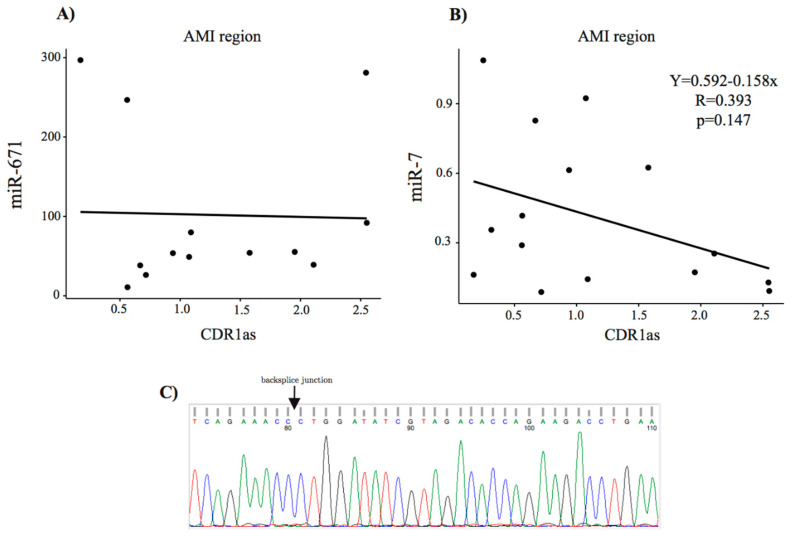
Correlation between CDR1as and miR-671 and miR-7, and the Sanger chromatogram. (**A**) Scatter plot of miR-671 expression versus CDR1as and (**B**) scatter plot of miR-7 versus CDR1as in the three treatment groups. (**C**) Sanger chromatogram shows the presence of a backsplice junction, indicated with a black arrow.

Furthermore, we explored whether miR-671 and miR-7 interact with CDR1as in the AMI region. Scatter plots show miR-671 and miR-7 versus CDR1as expression (Figure 8).

**Figure 9:**
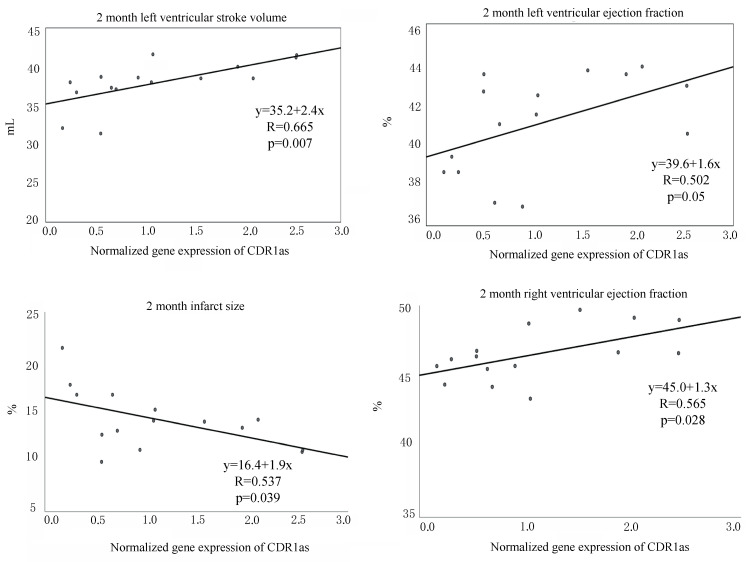
Correlation between normalized gene expression of CDR1as and left ventricular (LV) stroke volume (SV), ejection fraction (EF), infarct size, and right ventricular EF.

Additionally, we investigated the association between the tissue expression level of CDR1as and the LV and RV function and found a significant positive correlation between the CDR1as and LV and RV EF and LV SV and a negative correlation regarding infarct size, suggesting a direct beneficial effect of CDR1as on the heart function post-myocardial infarction (Figure 9).

## 4. Discussion

In our study, we demonstrated the successful detection of circRNA CDR1as in the infarcted myocardial area in pigs for the first time. Here, we show a moderate beneficial effect of the two natural compounds bufalin and lycorine for prevention of infarct-related LV remodeling, significantly less myocardial fibrosis in the remodeled ventricular wall and the increase in CDR1as expression. In the early phase of myocardial infarction, inflammation is the dominant myocardial process, and we did not want to interfere with the inflammation. The aim of the treatment was to inhibit fibrogenesis and adverse ventricular remodeling. As the fibrotic process starts later, we decided to start the treatments 7 days after the AMI onset. Interestingly, even in a small sample, we could show significant correlations between the infarct tissue upregulated CDR1as and improved LV and RV function and decreased infarct size.

The currently proposed mode of action for CDR1as is via binding of miR-7, thus acting as a miRNA sponge [19,29]. miR-7 was found to be overexpressed in stress-stimulated cardiomyocytes [30], and CDR1as overexpression in vivo was shown to significantly increase infarct size [21]. In contrast with the study of Geng et al., we found a beneficial effect of CDR1as on reducing infarct size, and a positive influence on LV and RV functions. The reason of the differences might be the chosen experimental species (mouse vs. pigs), the experimental settings (surgical permanent ligation of the LAD in mouse vs. closed-chest reperfused AMI in pigs), and the follow-up (24 h in mouse vs. 2 months in pigs). Since the porcine heart and cardiovascular physiology and pathophysiology have numerous similarities to humans, we may assume that our model is appropriate for translation to human ischemic heart disease [31]. Because we now showed that CDR1as is expressed in pig hearts, its therapeutic or biomarker character can therefore be further validated in several porcine ischemic heart disease models in vivo.

The assumption that CDR1as, acting as a miR-7 sponge, could potentially have great clinical significance is an important research target largely spurred by the high degree of sequence conservation between species. An almost perfect conservation was shown for miR-7 between species as evolutionarily distinct as annelids and humans [29]. Most of the studies published on the topic of CDR1as thus far were performed on murine models [21,32,33]. CDR1as is a relatively new target but has already been examined extensively in the field of oncology [19,28,34,35,36]. Another study attempted to isolate CDR1as in pig heart but failed [22]. One of the reasons for the failed detection could be the use of RNA-Seq, a technique that allows high volume sequence analysis and the discovery of novel targets, albeit with a somewhat reduced accuracy. This led us to choose PCR, with the hope of obtaining more sensitive measurements. To our knowledge, our study is the first to definitively describe the expression of CDR1as in porcine hearts. Our findings are further backed up by the Sanger sequencing results.

In line with previous in vitro and in vivo experiments [9], bufalin and lycorine showed a strong anti-fibrotic effect also in the translational and clinically relevant large animal pig model. Even if there was no significant change in expression levels on mRNA (MMP-9, COL1A1, COL3A1) or miRNA (miR-29) levels, PSR staining displayed a significantly larger fibrosis in the remote ischemia-non-affected myocardial regions. The difference between these findings can be explained by differences in the target biological and histological methods (quantification of mRNA or miRNA by qPCR or PSR stained fibrosis on protein level). However, we found high standard deviations for the gene expression analyses and also some functional parameters. Interestingly, the high standard deviation was mainly seen in groups treated with either BUF or LYC but not in the saline group. This could imply an individually variable response. In contrast with the hypertension-associated rodent cardiac fibrosis model, we here used an ischemia-induced ventricular remodeling model in large animals. The only modest beneficial effects of bufalin and lycorine towards cardiac function may be due to the relatively short treatment and follow-up periods.

## 5. Limitations

In this study, we did not investigate the myocardial expression levels of CDR1as, miR-7, and miR-671 of the healthy heart, because it should have required either a myocardial biopsy before AMI induction (which would influence the outcome of AMI) or including an additional normal healthy animal group.

## 6. Conclusions

Here, we demonstrated for the first time the successful detection of circRNA CDR1as in the infarcted myocardial area in pigs and showed a moderate beneficial effect of the two natural compounds bufalin and lycorine for prevention of infarct-related LV remodeling, less myocardial fibrosis in the remodeled ventricular wall, and the increase in CDR1as expression. The upregulated CDR1as in the infarcted tissue correlates significantly with the better LV and RV function and decreased infarct size.

## Supplementary Materials

The following are available online at https://www.mdpi.com/2218-273X/10/8/1180/s1, Table S1. Primers; Table S2. Baseline values and changes (**Δ**) in cardiac biomarkers (Troponin I and NT-proBNP) in the three groups between different time points; Figure S1: ALT, AST and ALP activity in serum at pre AMI, 14 days and 2 months after AMI; Figure S2: Serum Troponin I (pg/mL) and Pro-BNP (pg/mL) levels in BUF, LYC and SAL groups at baseline, after reperfused AMI and at different follow-ups; Figure S3: Microvascular obstruction (MVO, 3 days after AMI) and left ventricular end-diastolic volume (EDV); end-systolic volume (ESV) and stroke volume (SV) in the treatment groups; Figure S4: Cardiac MRI parameter of RV (3 days after AMI and 2 months FUP); Figure S5: Delta-EF and Delta-RVEF of individual animals Changes in ejection fraction (EF) and right ventricle EF (RVEF) of individual animals between 3 days after AMI and 2 months FU.
