# Supplementary material for: Association between Circular RNA CDR1as and Post-Infarction Cardiac Function in Pig Ischemic Heart Failure: Influence of the Anti-Fibrotic Natural Compounds Bufalin and Lycorine
Source: Biomolecules. 2020 Aug 14;10(8):1180. doi: 10.3390/biom10081180 (PMC7463784; doi:10.3390/biom10081180)
Supplement: Supplementary file 1 [file biomolecules-10-01180-s001.pdf]

Supplementary material

Supplementary methods

Haematoxylin-Eosin and Picrosirius Red staining

For Haematoxylin-Eosin and Picrosirius Red Staining, the myocardial tissue samples from the remote region of each animal were collected and preserved in 5% formaldehyde. The samples were then embedded in paraffin blocks and cut into 3 μM thick slices.

The slices were stained using a standard Haematoxylin-Eosin and Picrosirius Red stain, and the sections were examined under a 20x or 100x magnification and 8 representative photos were taken of each tissue. For calculation of the cell diameter, circumferences of 10 cell were manually outlined in Olympus software and the numeric length of the circumference was recorded. The process was repeated for each image. The corresponding diameters were calculated based on the calculated circumference. The values of the groups were expressed as mean ± SD.

For quantification of myocardial fibrosis, the Picrosirius Red stained tissue samples from the infarction remote region of each animal were examined under a 20x or 100x magnification and 8 representative photos of each sample were taken. All the images were taken using the same histogram settings on the Olympus IX83 microscope (Olympus, Tokyo, Japan) and they were all autofocused. The images were then processed using ImageJ software. The images were converted into RGB stack images. The fibrotic red areas were detected in the red channel using the same threshold values for all the samples. The total fibrous area was calculated numerically as the percentage of the total image area. The mean relative fibrous area and the SDs were calculated for each animal.

RNA isolation from tissue

Tissue samples of the infarcted myocardium were thawed at room temperature (rt) until RNAlater returned to a liquid state. Tissue (ca. 25 mg) was cut and put in CK 28 Precellys tube containing 1 mL Qiazol (Qiagen, Hilden, Germany). Using the Precellys 24 (program 5000:3x20 – 20 sec wait), tissue was homogenized. The liquid was transferred into 2 mL tubes (Eppendorf) and 200 μL chloroform was added. The solution was then vortexed, kept at rt for 2 minutes, followed by centrifugation (4 °C, 11600xg, 15 min). The upper, aqueous phase was collected and put in new 2 mL tubes (Eppendorf). The samples were placed into the QIAcube (Qiagen, Hilden, Germany) and total RNA including small RNAs was isolated using the miRNeasy Mini kit program. RNA concentration and purity was assessed using an UV-Vis spectrophotometer (Nanodrop, Thermo Fisher). The 260/280 ratio of the RNA samples were between 2.01 and 2.08.

cDNA synthesis for mRNA and miRNA

cDNA synthesis was done using the QuantiTect reverse transcription kit and the miScript II reverse transcription kit (Qiagen, Hilden, Germany). Method was performed according to the manufacturer's protocol.

Real-Time qPCR mRNA

The QuantiTect SYBR Green PCR kit (Qiagen, Hilden, Germany) was used for qPCR and method was performed according to the manufacturer's protocol. The QuantStudio 5 Real-Time PCR System (Thermo Fisher Scientific, Waltham, MA, USA) was used and following program selected: Hold stage, step one for 2 min. at 50 °C, step two for 15 min. at 95 °C, then 40 cycles of the following three PCR steps: Denaturation for 15 sec. at 95°C, annealing for 1 min. at 58-60 °C (depending on the primers) and extension (data collection) for 30 sec. at 72 °C. Melt curve stage (continuous) with step one 15 sec. at 95 °C, step two 1 min. at 60 °C and step 3 for 1 sec. at 95 °C. 8 ng of cDNA was used for the qPCR. Each sample was run in duplicates and no template controls (NTCs) were done for each qPCR to check for primer dimers or contamination. A dilution series was done to establish a standard curve for

each qPCR. The dilution series consisted of a cDNA sample pool and was made with RNase free water (1:8, 1:64, 1:512 and 1:4096). MicroAmp optical 96-well reaction plates (0.2 mL, applied biosystems) were sealed with an optical adhesive film (applied biosystems) and centrifuged for 30 s before running the qPCR. Normalized gene expression was calculated using the relative standard curve method. The geometric mean of ACTB and GAPDH was used as reference.

Real-Time qPCR miRNA

The following program was selected for miRNA qPCR: Hold stage, step one for 2 min, 50 °C, step two for 15 min. at 95 °C, then 50 cycles of the following three PCR steps: Denaturation for 15 sec. at 94 °C, annealing for 30 sec. at 55-60 °C (depending on the primers) and extension (data collection) for 30 sec. at 70 °C. Melt curve stage (continuous) with step one 15 sec. at 95 °C, step two 1 min. at 60 °C and step 3 for 1 sec. at 95 °C. All other qPCR steps and used materials were the same as in the mRNA protocol. The relative standard curve method was used to calculate normalized gene expression. Let-7a was used as reference.

The geometric mean of ACTB and GAPDH was used as a reference for COL1A1, COL3A1 and MMP-9. Let-7a was used as a reference for miR-29a.

Name	Forward (5′-3′)	Reverse (5′-3′):
ACTB (Microsynth)<br>*Housekeeping gene*	TCA ACA CCC AGC CAT GTA C	CTC CGG AGT CCA TCA CGA TG
GAPDH (Microsynth)<br>*Housekeeping gene*	GGT CGG AGT GAA CGG ATT T	ATG TAG TGG AGG TCA ATG AAG G
MMP-9 (Microsynth)	CGC GAG ACC TAC GAA CCA AT	TTT CTG TCG GTC ATC TCG GC
COL1A1 (Eurofins Genomics)	CTG GCC TCC CTG TGA AG	CAC CCT TAG CAC CAA CAG CA
COL3A1 (Eurofins Genomics)	GCT CCC ATC TTG GTC AGT CC	GCC ACC AGT AGG GCA TGA TT
CDR1as No.1 (Eurofins Genomics)	TGT CTC CAG TGT ACT GGT C	AAG ACA TGG ATT GTC CGG AA
CDR1as No.2 (Eurofins Genomics)	CCA GCC TAC CCA TGT CTT CC	CCT TTG TTG GAA GAC GCA CC
CDR1as No.3 (Eurofins Genomics)	CCA GGT CTT CCA CTC AGT CC	AGG AAG ACC CGA ATT GTC CG
miR-671-5p (Eurofins Genomics)	5′ CGT ATA GGA AGC CCT GGA G 3′	
miR-29a (Microsynth)	5′ CGG ACC TAG CAC CAT CTG AA 3′	
let-7a (Microsynth)<br>*Housekeeping gene*	5′ GCA GTG AGC TAG TAG GTT GT 3′	

Supplementary Table S1. Primers

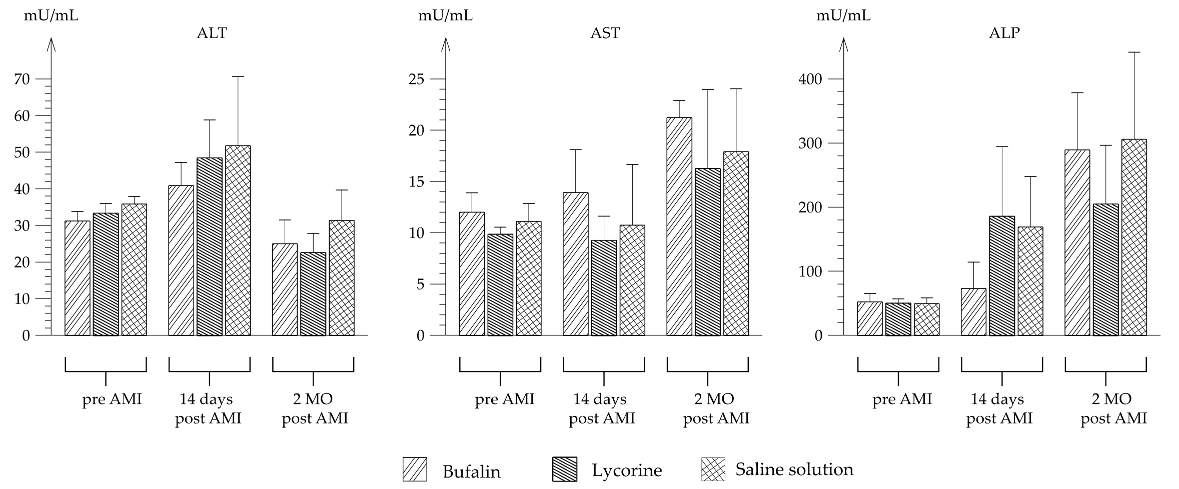

Supplementary Figure S1: ALT, AST and ALP activity in serum at pre AMI, 14 days and 2 months after AMI. Vertical bars show the average of each group, error bars represent standard deviation. Each group: n = 5

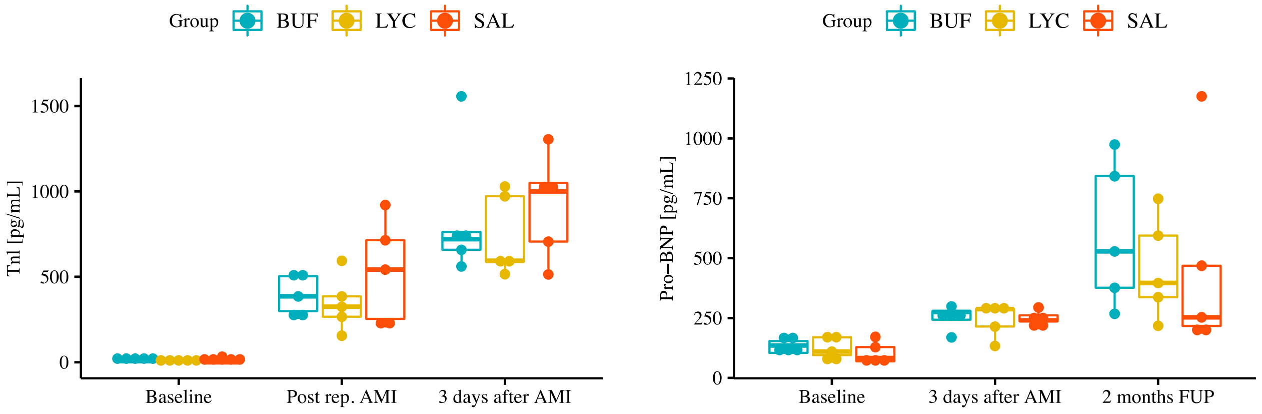

Supplementary Figure S2: Serum Troponin I (pg/mL) and Pro-BNP (pg/mL) levels in BUF, LYC and SAL groups at baseline, after reperfused AMI and at different follow-ups. Each group: n = 5

Groups	Baseline	ΔTnI-Base-postAMI	ΔTnI Base-FUP	ΔTnI post-AMI-FUP
BUF	18.2±16.34	373.5±127.8	833.9±396.4	460.4±494.7
LYC	9.4±8.79	336.0±163.4	730.8±241.1	394.8±185.1
SAL	8.4±13.49	518.7±295.2	906.9±297.4	388.2±268.4
ttest BUF_SAL	0.383	0.342	0.750	0.782
ttest LYC_SAL	0.91	0.261	0.334	0.965

Groups	Baseline	ΔNT-proBNP-Base-3D	ΔNT-proBNP-Base-FUP	ΔNT-proBNP 3D-FUP
BUF	134.6±30.69	118.8±57.7	463.4±319.7	344.6±332.3
LYC	122.24±42.38	121.4±83.0	336.6±175.8	215.1±224.9
SAL	103.45±40.84	143.7±61.2	356.5±427.2	212.8±391.5
ttest BUF_SAL	0.26	0.527	0.666	0.582
ttest LYC_SAL	0.54	0.642	0.926	0.991

Supplementary Table S2. Baseline values and changes (Δ) in cardiac biomarkers (Troponin I and NT-proBNP) in the three groups between different time points

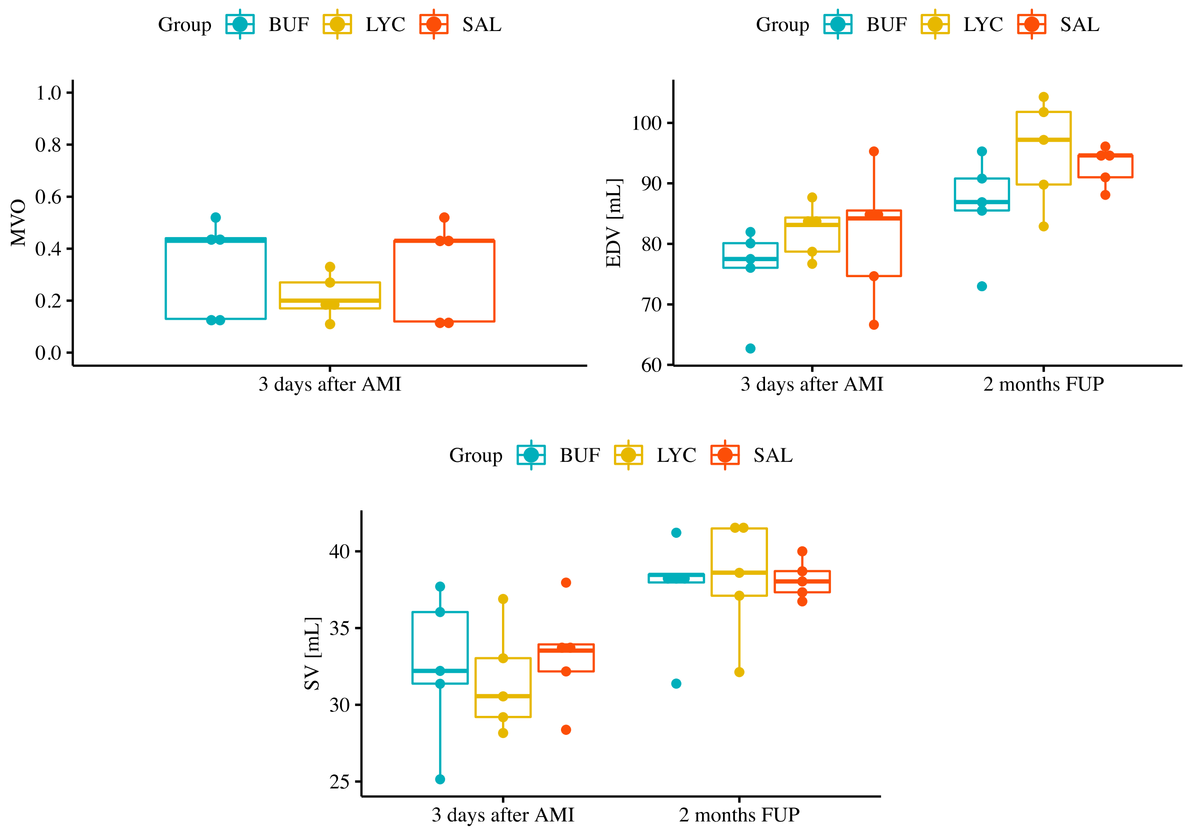

Supplementary Figure S3: Microvascular obstruction (MVO, 3 days after AMI) and left ventricular end-diastolic volume (EDV); end-systolic volume (ESV) and stroke volume (SV) in the treatment groups.

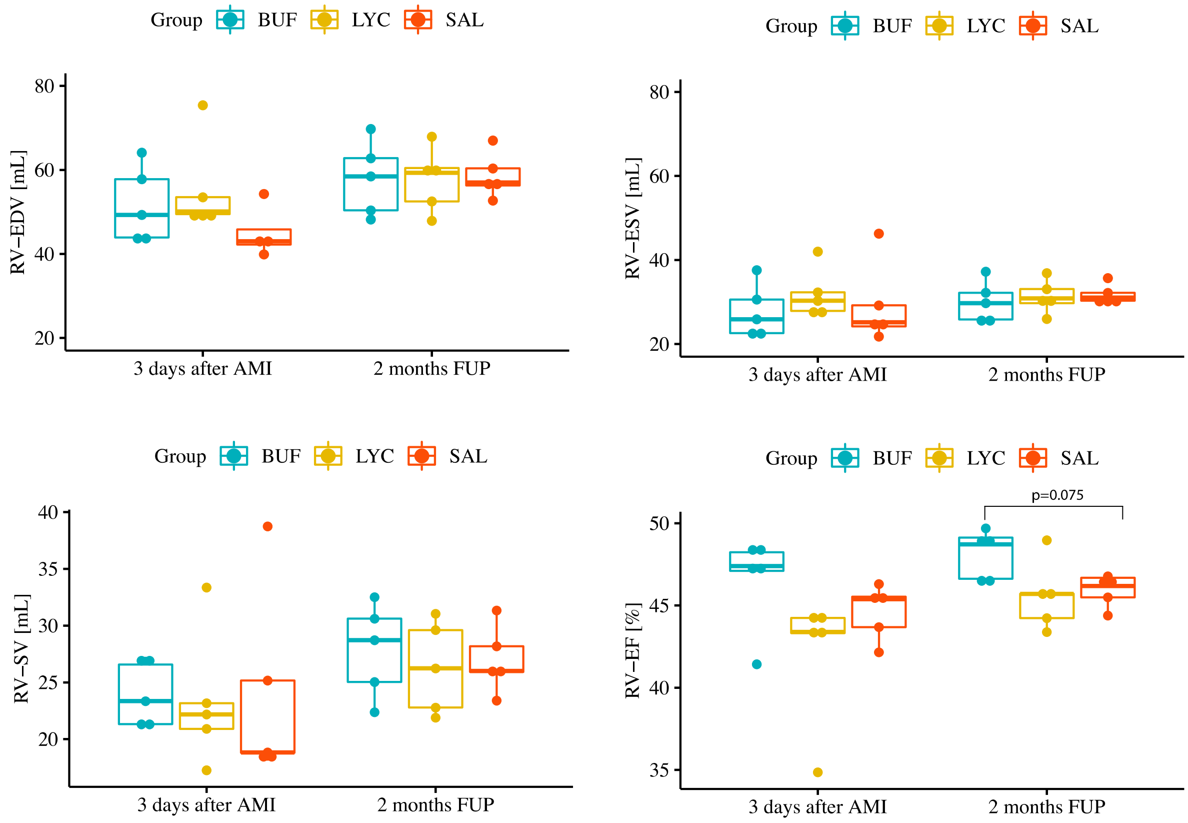

Supplementary Figure S4: Cardiac MRI parameter of RV (3 days after AMI and 2 months FUP)
RV = right ventricular; EDV = end-diastolic volume; ESV = end-systolic volume SV = stroke volume; EF = ejection fraction.

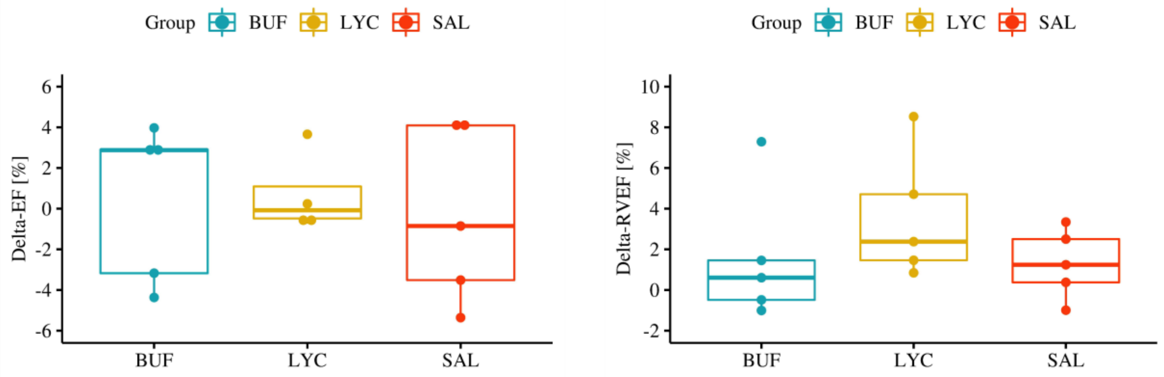

Supplementary Figure S5: Delta-EF and Delta-RVEF of individual animals
Changes in ejection fraction (EF) and right ventricle EF (RVEF) of individual animals between 3 days after AMI and 2 months FUP
